# Supplementary material for: Development of a computer-aided design software for the quantitative evaluation of aesthetic damage
Source: PLoS One. 2019 Dec 18;14(12):e0226322. doi: 10.1371/journal.pone.0226322 (PMC6919621; doi:10.1371/journal.pone.0226322)
Supplement: S1 Code site — 1. (ZIP) [file pone.0226322.s001.zip › [EESC_jr][Projeto]Relatorio_07_21/reports/templates_zip/gentelella-master/vendors/bootstrap-daterangepicker/website/index.html]

Date Range Picker for Bootstrap


- Date Range Picker

- Website Goodies
- Improvely
- W3Counter
- Visitor Boost

# Date Range Picker

A JavaScript component for choosing date ranges.
  
Designed to work with the Bootstrap CSS framework.

View on GitHub
 
Download ZIP

- Usage
- Examples
  - Date Range Picker
  - Date and Time
  - Single Date Picker
  - Predefined Ranges
  - Input Initially Empty
- Configuration Generator
- Options, Methods & Events
- License

Originally built for reporting at Improvely,
the Date Range Picker can be attached to any webpage element to pop up two calendars
for selecting dates, times, or from predefined ranges like "Last 30 Days".

## Usage

Date Range Picker relies on Bootstrap, jQuery and Moment.js.
Include the required scripts and stylesheet in your page:

Then attach the picker to the element you want to trigger it:

---

You can customize Date Range Picker with options, and
get notified when the user chooses new dates by providing a callback function.

## Examples

### Date Range Picker

The Date Range Picker is attached to a text input. It will use the current
value of the input to initialize, and update the input if new dates are chosen.

#### Demo:

### Date and Time

The Date Range Picker can also be used to select times. Hour, minute and (optional)
second dropdowns are added below the calendars. An option exists to set the increment
count of the minutes dropdown to e.g. offer only 15-minute or 30-minute increments.

#### Demo:

### Single Date Picker

The Date Range Picker can be turned into a single date picker widget with only
one calendar. In this example, dropdowns to select a month and year have also
been enabled at the top of the calendar to quickly jump to different months.

#### Demo:

### Predefined Ranges

This example shows the option to predefine date ranges that
the user can choose from a list.

#### Demo:

### Input Initially Empty

If you're using a date range as a filter, you may want to attach a picker to an
input but leave it empty by default. This example shows how to accomplish that
using the `autoUpdateInput` setting, and the `apply` and
`cancel` events.

#### Demo:

## Configuration Generator

parentEl

startDate

endDate

minDate

maxDate

autoApply

singleDatePicker

showDropdowns

showWeekNumbers

showISOWeekNumbers

timePicker

timePicker24Hour

timePickerIncrement (in minutes)

timePickerSeconds

dateLimit (with example date range span)

ranges (with example predefined ranges)

locale (with example settings)

linkedCalendars

autoUpdateInput

alwaysShowCalendars

opens

right
left
center

drops

down
up

buttonClasses

applyClass

cancelClass

#### Your Date Range Picker

#### Configuration

## Options

- `startDate` (Date object, moment object or string) The start of the initially selected date range
- `endDate`: (Date object, moment object or string) The end of the initially selected date range
- `minDate`: (Date object, moment object or string) The earliest date a user may select
- `maxDate`: (Date object, moment object or string) The latest date a user may select
- `dateLimit`: (object) The maximum span between the selected start and end dates. Can have any property you can add to a moment object (i.e. days, months)
- `showDropdowns`: (boolean) Show year and month select boxes above calendars to jump to a specific month and year
- `showWeekNumbers`: (boolean) Show localized week numbers at the start of each week on the calendars
- `showISOWeekNumbers`: (boolean) Show ISO week numbers at the start of each week on the calendars
- `timePicker`: (boolean) Allow selection of dates with times, not just dates
- `timePickerIncrement`: (number) Increment of the minutes selection list for times (i.e. 30 to allow only selection of times ending in 0 or 30)
- `timePicker24Hour`: (boolean) Use 24-hour instead of 12-hour times, removing the AM/PM selection
- `timePickerSeconds`: (boolean) Show seconds in the timePicker
- `ranges`: (object) Set predefined date ranges the user can select from. Each key is the label for the range, and its value an array with two dates representing the bounds of the range
- `opens`: (string: 'left'/'right'/'center') Whether the picker appears aligned to the left, to the right, or centered under the HTML element it's attached to
- `drops`: (string: 'down' or 'up') Whether the picker appears below (default) or above the HTML element it's attached to
- `buttonClasses`: (array) CSS class names that will be added to all buttons in the picker
- `applyClass`: (string) CSS class string that will be added to the apply button
- `cancelClass`: (string) CSS class string that will be added to the cancel button
- `locale`: (object) Allows you to provide localized strings for buttons and labels, customize the date display format, and change the first day of week for the calendars
- `singleDatePicker`: (boolean) Show only a single calendar to choose one date, instead of a range picker with two calendars; the start and end dates provided to your callback will be the same single date chosen
- `autoApply`: (boolean) Hide the apply and cancel buttons, and automatically apply a new date range as soon as two dates or a predefined range is selected
- `linkedCalendars`: (boolean) When enabled, the two calendars displayed will always be for two sequential months (i.e. January and February), and both will be advanced when clicking the left or right arrows above the calendars. When disabled, the two calendars can be individually advanced and display any month/year.
- `parentEl`: (string) jQuery selector of the parent element that the date range picker will be added to, if not provided this will be 'body'
- `isInvalidDate`: (function) A function that is passed each date in the two
  calendars before they are displayed, and may return true or false to indicate whether
  that date should be available for selection or not.
- `isCustomDate`: (function) A function that is passed each date in the two
  calendars before they are displayed, and may return a string or array of CSS class names
  to apply to that date's calendar cell.
- `autoUpdateInput`: (boolean) Indicates whether the date range picker should
  automatically update the value of an `<input>` element it's attached to
  at initialization and when the selected dates change.
- `alwaysShowCalendars`: (boolean) Normally, if you use the `ranges`
  option to specify pre-defined date ranges, calendars for choosing a custom date range are not shown until the user clicks "Custom Range". When this option is set to true, the calendars for choosing a custom date range are always shown instead.

## Methods

You can programmatically update the `startDate` and `endDate`
in the picker using the `setStartDate` and `setEndDate` methods.
You can access the Date Range Picker object and its functions and properties through
data properties of the element you attached it to.

  

- `setStartDate(Date/moment/string)`: Sets the date range picker's currently selected start date to the provided date
- `setEndDate(Date/moment/string)`: Sets the date range picker's currently selected end date to the provided date

**Example usage:**

## Events

Several events are triggered on the element you attach the picker to, which you can listen for.

- `show.daterangepicker`: Triggered when the picker is shown
- `hide.daterangepicker`: Triggered when the picker is hidden
- `showCalendar.daterangepicker`: Triggered when the calendar(s) are shown
- `hideCalendar.daterangepicker`: Triggered when the calendar(s) are hidden
- `apply.daterangepicker`: Triggered when the apply button is clicked,
  or when a predefined range is clicked
- `cancel.daterangepicker`: Triggered when the cancel button is clicked

Some applications need a "clear" instead of a "cancel" functionality, which can be achieved by changing the button label and watching for the cancel event:

  

While passing in a callback to the constructor is the easiest way to listen for changes in the selected date range, you can also do something every time the apply button is clicked even if the selection hasn't changed:

## License

The MIT License (MIT)

Copyright (c) 2012-2015 Dan Grossman

Permission is hereby granted, free of charge, to any person obtaining a copy of this software and associated documentation files (the "Software"), to deal in the Software without restriction, including without limitation the rights to use, copy, modify, merge, publish, distribute, sublicense, and/or sell copies of the Software, and to permit persons to whom the Software is furnished to do so, subject to the following conditions:

The above copyright notice and this permission notice shall be included in all copies or substantial portions of the Software.

THE SOFTWARE IS PROVIDED "AS IS", WITHOUT WARRANTY OF ANY KIND, EXPRESS OR IMPLIED, INCLUDING BUT NOT LIMITED TO THE WARRANTIES OF MERCHANTABILITY, FITNESS FOR A PARTICULAR PURPOSE AND NONINFRINGEMENT. IN NO EVENT SHALL THE AUTHORS OR COPYRIGHT HOLDERS BE LIABLE FOR ANY CLAIM, DAMAGES OR OTHER LIABILITY, WHETHER IN AN ACTION OF CONTRACT, TORT OR OTHERWISE, ARISING FROM, OUT OF OR IN CONNECTION WITH THE SOFTWARE OR THE USE OR OTHER DEALINGS IN THE SOFTWARE.

## Comments

Please enable JavaScript to view the comments powered by Disqus.


Copyright © 2015 Awio Web Services LLC.
 
Developed and maintained by Dan Grossman.
